# Seeking treatment for traumatic dental injuries in schoolchildren: A multilevel analysis

**DOI:** 10.1590/0103-6440202305555

**Published:** 2023-12-22

**Authors:** Veruska Medeiros Martins Bernardino, Larissa Chaves Morais de Lima, Luiza Jordânia Serafim de Araújo, Érick Tássio Barbosa Neves, Fernanda de Morais Ferreira, Ana Flávia Granville-Garcia

**Affiliations:** 1. Graduate program in Dentistry, State University of Paraiba (UEPB), Campina Grande-PB, Brazil; 2 Graduate program in Dentistry, Federal University of Minas Gerais (UFMG), Belo Horizonte-MG, Brazil

**Keywords:** traumatic dental injury, treatment, schools, health literacy, child

## Abstract

To investigate individual and contextual factors associated with seeking treatment for traumatic dental injuries. A cross-sectional study was conducted with 739 pairs of guardians and children. Guardians answered a questionnaire addressing sociodemographic characteristics, seeking treatment for a tooth injury in the child, and the Oral Health Literacy-Adult Questionnaire. Descriptive analysis and unadjusted and adjusted multilevel Poisson regression analysis (p<0.05). The prevalence of seeking treatment for traumatic dental injury was 44.7%. Sociodemographic variables associated were white skin of the child (PR = 1.30; 95% CI: 1.08-1.76), mother’s age older than 35 years (PR = 1.70; 95% CI: 1.50-1.90), married parents (PR = 1.93; 95% CI: 1.70-2.23), guardians with more than eight years of schooling (PR = 2.00; 95% CI: 1.76-2.23), with adequate oral health literacy (PR = 3.33; 95% CI: 3.01-3.62) and the daily use of electronic devices by the child (PR = 1.26; 95% CI: 1.02-1.50). On the contextual level, attending a private school (PR = 1.77; 95% CI: 1.02-3.05) and the number of primary care units with oral health teams in the school district (PR = 1.78; 95% CI: 1.12-2.38) remained associated. Among the children who suffered TDI, adequate oral health literacy on the part of the parents stood out among the factors associated with seeking treatment.

## Resumo

Investigar fatores individuais e contextuais associados à busca de tratamento para lesões dentárias traumáticas. Foi realizado um estudo transversal com 739 pares de responsáveis e crianças. Os responsáveis responderam a um questionário abordando características sociodemográficas, busca por tratamento para traumatismo dentário na criança e o Questionário de alfabetização em saúde bucal para adulto. Análise descritiva e análise de regressão de Poisson multinível não ajustada e ajustada (p<0,05) foram realizadas. A prevalência de busca de tratamento para lesão dentária traumática foi de 44,7%. As variáveis sociodemográficas associadas foram a pele branca da criança (RP = 1,30; IC 95%: 1,08-1,76), idade da mãe maior que 35 anos (RP = 1,70; IC 95%: 1,50-1,90), os responsáveis casados (RP = 1,93; IC 95%: 1,70-2,23), com mais de oito anos de estudo (RP = 2,00; IC 95%: 1,76-2,23), com alfabetização adequada em saúde bucal (RP = 2,00; IC 95%: 1,76-2,23) e uso diário de dispositivos eletrônicos pela criança (RP = 1,26; IC 95%: 1,02-1,50). No nível contextual, frequentar uma escola particular (PR = 1,77; IC 95%: 1,02-3,05) e a quantidade de unidades básicas de saúde com equipes de saúde bucal no distrito sanitário da escola (PR = 1,78; IC 95%: 1,12-2,38) permaneceram associados. Entre as crianças que sofreram TDI, a alfabetização em saúde bucal adequada dos pais foi um fator que se destacou na busca por tratamento.

## Introduction

Traumatic dental injuries (TDIs) constitute a public health problem due to the high prevalence and negative impacts on physical, social, and psychological well-being as well as the high cost of treatment ^1^. The age group with the highest occurrence is eight to ten years of age, in which the prevalence of TDI ranges from 4.6% to 14% ^2^,^3^. Even mild TDIs can have sequelae, which can be minimized by periodic follow-up appointments involving clinical and radiographic exams and timely treatment ^4^ in cases of pulp necrosis or pulp canal obliteration, root resorption, and rupture of the marginal gingiva and bone ^5^.

Treatment for TDI is often neglected, which can exert an impact on the oral health-related quality of life of children ^6^. However, predictive factors of seeking treatment for TDI are largely unknown. Knowledge of individual and contextual factors that may be associated with seeking treatment could assist in the planning of public policies that contribute to better health practices.

The family environment influences health-related practices and the mother is considered the main caregiver in numerous cultures ^7^. Thus, a mother's schooling exerts an influence on the oral health of her children and seeking health care services ^8^. These aspects underscore the importance of the involvement of the family in the prevention of sequelae from TDIs.

Another aspect that has recently been the target of investigation is the influence of the internet in the dissemination of knowledge and the promotion of self-care among children and adolescents. The use of electronic devices with internet access has facilitated access to information on health ^8^. The ability to seek and understand knowledge available through electronic devices and apply this knowledge to decision-making is an increasingly present skill in the lives of individuals ^9^. Indeed, the mobile app denominated ToothSOS may be an important mechanism for seeking the timely treatment of TDI. The International Association of Dental Traumatology recommends this app and its dissemination by dental professionals is important ^10^. Moreover, access to this resource could assist pediatric dentists in sensitizing children to seek treatment for TDIs.

One point that has been explored in the literature is the association between oral health literacy and health outcomes ^11^. However, no previous studies have investigated the influence of this variable on seeking treatment for TDIs. Oral health literacy regards (OHL) the acquisition, understanding, and use of information to favor decision-making for the benefit of oral health. Lower OHL translates to a lower understanding of information and less use of dental services ^11^. The conceptual hypothesis of the present study is that an adequate level of OHL on the part of parents/guardians influences the seeking of treatment for TDIs in children eight to ten years of age.

With regards to contextual factors, the type of school and the quantity of Family Health Units (primary care modality) with oral health teams in the school district have not been investigated in terms of seeking treatment for TDI in children eight to ten years of age. These places, which are frequented by children, could be the target of health education programs. Moreover, health promotion in these settings reaches families and can sensitize parents/guardians to seek treatment for TDIs ^12^.

The present study aimed to investigate individual and contextual factors associated with seeking treatment for traumatic dental injuries in schoolchildren eight to ten years of age.

## Methods

### Study design and selection of sample

A cross-sectional study was conducted with 739 schoolchildren eight to ten years of age at public and private schools in the city of Campina Grande, Brazil, following approval from the Human Research Ethics Committee of Universidade Estadual da Paraíba (certificate number: 10514619.2.0000.5187). Children who wore orthodontic appliances, and those with special physical, behavioral, and sensorial needs reported by parents/guardians or teachers were not included in the study. Children who had no physical, sensorial, or behavioral problems and those not in needs of special education or attention reported by teachers were included.

The sample size was calculated for analytical comparison studies between two independent proportions with the aid of the G* Power software program, version 3.1 (Franz Faul, Universitat Kiel, Germany), considering a 95% significance level and a 5% acceptable rate of error. Estimates for the sample calculation were based on the results of the pilot study, in which the prevalence of children who sought treatment for TDI whose parents/guardians had low and high OHL was respectively 32% and 44.2%. Using these data, the minimum sample size was determined to be 390 children. An effect size of 1.6 was applied to increase the sample variation and 20% was added to compensate for possible dropouts, leading to an estimated final sample of 780 children eight to ten years of age.

### Training and calibration

Training for the diagnosis of TDI was conducted in two steps: theory and practice. In the first step, an expert in the field regarding the diagnostic criteria proposed by Andreasen ^13^ provided theoretical information to the examiners. The second step involved the diagnosis of TDI from images using a video projector. Four examiners performed next, clinical examinations of TDI. For such, 40 schoolchildren were examined on two occasions with a seven-day interval between examinations. The Kappa statistic was used for the determination of intra-examiner (K = 0.89-0.90) and inter-examiner (K = 0.81-0.88) agreement.

### Pilot study

A pilot study was performed with 40 children from public and private schools to test the study methods. The results revealed that the methods were adequate. The children who participated in this step were not included in the main study.

Individual determinants

The following sociodemographic characteristics were collected: child's sex and age, mother's age (categorized by the median), guardian is schooling (≤ eight years of study or > eight years of study), and guardian's marital status. Parents/guardians reported whether they had ever sought dental treatment/care for a tooth injury in their child. Seeking dental care in cases of TDI is important so that children and parents can be counseled about adequate care for healing. In some, cases subsequent treatments with specialized secondary interventions may be necessary ^5^,^14^,^15^. Before the clinical examinations, the children reported whether they used electronic devices, such as a cellular phone, tablet, or computer.

The Oral Health Literacy-Adult Questionnaire (OHL-AQ) was also sent to the parents/guardians for the assessment of OHL. This is a self-administered questionnaire with 17 items distributed among four sections: reading comprehension, numeracy, listening, and decision-making. Reading comprehension consists of incomplete sentences on oral health knowledge. The listening section consists of two items on post-extraction instructions. The decision-making section has five items on common oral health problems and medical history. Correct answers are scored one point and incorrect answers are scored zero points. The sum of the item scores furnishes a total between zero and 17 points, which enables the classification of OHL as inadequate (0-9 points), marginal (10 and 11 points), or adequate (> 12 points). The OHL-AQ has been validated for use in the Brazilian population ^16^.

### Clinical examination

Examinations were performed at the schools at predefined times during the shift (morning or afternoon) in which the student was enrolled in a separate room with the child in the sitting position. The examiners used individual protective equipment and a portable LED light positioned on the head (Petzl Zoom head lamp, Petzl America, Clearfield, UT, USA). The intraoral examinations were performed with the aid of sterile mouth mirrors (PRISMA, São Paulo, SP, Brazil), sterile WHO probes (OMS-621-Trinity, Campo Mourão, PA, Brazil), and gauze to dry the teeth. TDI was diagnosed using the classification criteria proposed by Andreasen ^13^: absence of trauma, enamel fracture, enamel + dentin fracture, complicated crown fracture, extrusive luxation, lateral luxation, intrusive luxation, and avulsion. Tooth discoloration, combined trauma, and restoration due to TDI were also investigated. Based on these clinical situations, TDI was dichotomized as present or absent and also classified as uncomplicated (involving only enamel or enamel + dentin) or complicated (other TDIs). Only the maxillary and mandibular incisors and canines were inspected for the determination of TDI.

### Contextual determinants

The contextual variables of interest were the type of school (public or private) and the number of primary care units with an oral health team in the school district. The city in which the study was conducted is divided into six health districts. The health establishments that make up the primary care network in Brazil are called primary care units, with Family Health Teams (physicians, nurses, and community health agents) and oral health teams composed of dentists and oral health assistants or technicians. These primary care units are located in the health districts, which are areas that have populations with the same epidemiological and social characteristics as well as the same health care resources ^17^.

### Statistical analysis

Data analysis was performed with the aid of SPSS Statistics (SPSS for Windows, version 25.0, IBM Inc, Armonk, NY, USA). Descriptive statistics were performed with the calculation of absolute and relative frequencies for the characterization of the sample, followed by the unadjusted analysis with the level of significance set at 5% (p < 0.05). Unadjusted and adjusted multilevel Poisson regression analyses were then performed to describe associations between the independent variables and outcomes.

In the first step of the multilevel analysis, an unconditional (null) model was run to estimate the variability in the data before the incorporation of the individual and contextual characteristics (18). Independent variables with a p-value < 0.20 in the unadjusted Poisson regression analysis were incorporated into the second (individual variables) and third (contextual variables) multilevel models. Variables with a p-value < 0.05 after the adjustments remained in the final model.

## Results

The final sample was composed of 739 schoolchildren eight to ten years of age. The response rate was 94.7%. Losses (n = 41) occurred due to absences of the children on the three occasions for which the examinations were scheduled (n = 32) and refusals to participate in the study (n = 9). Table 1 displays the absolute and relative frequencies of the variables of interest. The prevalence of TDI was 16.2% and the prevalence of seeking treatment in such cases was 44.7%.

In the unadjusted analysis (Table 2), the individual variables associated with seeking treatment for TDI were children with white skin color, mothers older than 35 years, parents/guardians with more than eight years of study and adequate oral health literacy on the part of parents/guardians. The contextual variables associated with the outcome were attending private school and number of primary care units with an oral health team in the school district.

**Table 1:** Occurrence of traumatic dental injury, having sought treatment and independent variables in children eight to ten years of age.

	N	%
Individual variables		
Traumatic dental injury		
Yes	120	16.2
No	619	83.8
Sought treatment for traumatic dental injury		
Yes	84	44.7
No	104	55.3
Sex		
Male	367	49.7
Female	372	50.3
Skin color		
White	255	34.6
Non-White	483	65.4
Child’s age		
8 years	269	36.4
9 years	240	32.5
10 years	230	31.1
Mother’s age (dichotomized by median)		
≤ 35 years	384	52.7
>35 years	345	47.3
Schooling of parents/guardians		
≤ 8 years of study	317	43.0
> 8 years of study	420	57.0
Marital status of parents/guardians		
Not married	285	38.6
Married	453	61.3
Oral health literacy		
Insufficient	255	34.5
Marginal	275	37.2
Adequate	209	28.3
Familiar adaptability		
Chaotic	514	69.6
Structured	225	30.4
Daily use of electronic devices		
Yes	450	61.0
No	288	39.0
Contextual variables		
Type of school	349	47.2
Public		
Private	390	52.8
Number of oral health teams in school district (mean ± SD)	5.9 (1.6)	
Monthly income (R$) of school neighborhood (mean ± SD)	1201 (± 439)	

**Table 2:** Unadjusted Poisson regression analysis for complex samples of independent and contextual variables according to seeking treatment in children eight to ten years of age.

Sought Treatment for Traumatic Dental Injury				
Variables	Yes	No	p-value	Unadjusted PR
	n (%)	n (%)		
Sex				
Male	52 (54.2)	44 (45.8)	0.94	0.98 (0.63-1.52)
Female	32 (34.8)	60 (65.2)		1
Skin color				
White	41 (60.3)	27 (39.7)	0.06	1.53 (0.99-2.34)
Non-White	43 (36.1)	76 (63.9)		1
Mother’s age				
>35 years	49 (55.7)	39 (44.3)	0.03	1.63 (1.03-2.57)
≤35 years	35 (36.5)	61 (63.5)		1
Guardian’s marital status				
Married	54 (46.2)	63 (53.8)	0.02	1.71 (1.07-2.73)
Not married	30 (42.3)	41 (57.7)		1
Guardian’s schooling				
>8 years of study	49 (48.0)	53 (52.0)	<0.01	1.93 (1.19-3.13)
≤8 years of study	34 (40.5)	50 (59.5)		1
Daily use of electronic devices				
Yes	15 (60.2)	10 (40.0)	0.10	1.05 (0.13-0.79)
No	69 (42.6)	93 (57.4)		1
Family adaptability				
Chaotic	61 (45.5)	73 (54.5)	0.70	1.89 (0.48-1.62)
Structured	23 (42.6)	31 (57.4)		1
Oral health literacy				
Adequate	35 (53.0)	31 (47.0)	<0.01	2.91 (1.66-5.11)
Marginal	27 (50.9)	26 (49.1)	0.02	1.42 (0.73-2.78)
Insufficient	22 (31.9)	47 (68.1)		1
Type of school				
Private	41 (48.3)	58 (51.7)	0.01	2.51 (1.18-5.34)
Public	43 (41.4)	46 (58.6)		1
Oral health teams (mean ± SD)	7.0 (1.5)	5.6 (1.4)	0.02	2.02 (1.10-3.62)
Monthly income (R$) of school neighborhood (mean ± SD)	1163 (419)	1.042 (444)	0.50	1.21 (0.68-2.53)

In the multilevel Poisson regression analysis (Table 3), the individual variables associated with traumatic dental injury were white children (PR = 1.30; 95% CI: 1.08-1.76), mothers aged older than 35 years (PR = 1.70; 95% CI: 1.50-1.90), parents/guardians with more than eight years of schooling (PR = 2.00; 95% CI: 1.76-2.23), married parents (PR = 1.93; 95% CI: 1.70-2.23), the daily use of electronic devices by the children (PR = 1.26; 95% CI: 1.02-1.50) and parents/guardians with adequate oral health literacy (PR = 3.33; 95% CI: 3.01-3.62). On the contextual level, attending a private school (PR = 1.77; 95% CI: 1.02-3.05) and the number of primary care units with oral health teams in the school district (PR = 1.78; 95% CI: 1.12-2.38) remained associated with seeking treatment for traumatic dental injuries.

**Table 3:** Multilevel analysis of individual/contextual variables and seeking treatment for traumatic dental injury in children eight to ten years of age.

	Model 1 (“null”)	Model 2		Model 3	
	PR (95% CI)	p-value	PR (95% CI)	p-value	PR (95% CI)
Intercept	0.24 (0.11-0.37)		0.87 (0.64-2.38)		0.56 (0.17-3.34)
Individual variables					
Sex					
Male	-	-	-	-	-
Female	-	-	-	-	-
Skin color					
White	-	0.01	1.57 (1.39-1.75)	<0.01	1.30 (1.08-1.76)
Non-White	-	-	1	-	1
Mother’s age					
>35 years	-	0.01	1.19 (1.05-1.37)	0.01	1.70 (1.50-1.90)
≤35 years	-	-	1	-	1
Schooling of parents/guardians					
>8 years of study	-	0.01	1.44 (1.21-1.67)	0.01	2.00 (1.76-2.23)
≤8 years of study	-	-	1	-	1
Marital status of parents/guardians					
Married	-	<0.01	1.39 (1.19-1.58)	0.03	1.93 (1.70-2.13)
Not married	-	-	1	-	1
Daily use of electronic devices					
Yes	-	0.01	1.71 (1.45-1.97)	<0.01	1.26 (1.02-1.50)
No	-		1	-	1
Family adaptability					
Chaotic	-	-	-	-	-
Structured	-	-	-	-	-
Oral health literacy					
Adequate		<0.01	2.76 (2.55-2.97)	<0.01	3.33 (3.01-3.62)
Marginal			1.31 (1.19-1.54)		2.89 (2.64-3.14)
Insufficient			1		1
Contextual variables					
Type of school					
Private	-	-	-	0.03	1.77 (1.02-3.05)
Public	-	-	-		1
Oral health teams(mean ± SD)	-	-		0.01	1.78 (1.12-2.38)1
Monthly income of school neighborhood (mean ± SD)	-	-	-	-	-
Deviance (-2loglikelihood)	5703.33		5274.64		4989.71

## Discussion

In the present study, children with white skin color, mothers older than 35 years of age, parents/guardians with more than eight years of schooling, married parents/guardians, the use of electronic devices by the children, and adequate OHL on the part of parents/guardians were individual predictors of seeking treatment for TDI. Attending private schools and the number of primary care units with an oral health team in the school district were contextual determinants of seeking treatment for TDI. This is the first study to analyze the influence of OHL on seeking treatment for TDI in the mixed dentition phase. The conceptual hypothesis that adequate OHL on the part of parents/guardians is associated with the prevalence of seeking treatment for TDI was confirmed.

The present findings are important to the planning of public health programs and policies directed at encouraging parents/guardians to seek oral health services following the occurrence of TDIs. Such actions could diminish social disparities in seeking treatment, as differences are found in treatment for children of different countries and different social classes ^19^. These data show an opportunity for health administrators to intensify health education actions at schools and primary care units not only for the prevention of TDI but also for the treatment and follow-up of cases.

The parents of all children examined for the diagnosis of TDI in the present study answered a question on seeking dental treatment in a previous episode of TDI. This explains the higher prevalence of seeking treatment compared to data from other studies that did not analyze previous episodes of TDI ^8^, ^12^.

Less than half of the children who had an episode of TDI were taken to an oral health service. As reported in other studies, most TDIs are uncomplicated fractures that do not require immediate treatment but should have follow-up for the prevention of future sequelae ^4^, which underscores the need for counseling on seeking treatment in such cases.

Socioeconomic factors were predictors of seeking treatment for TDI among schoolchildren in the mixed dentition phase. Having white skin color was a predictor of seeking treatment. A previous study found this same result in a similar population ^20^, possibly because studies demonstrate greater access to services by individuals with white skin in Brazil ^21^. Children whose mothers were more than 35 years of age were more likely to have sought treatment, possibly because older mothers have more experience and perceive the needs of their children better ^22^. Guardians with more than eight years of schooling sought treatment for TDI more, demonstrating that a higher level of schooling on the part of parents/guardians leads to a greater likelihood of seeking oral health services ^8^,^20^.

Seeking treatment was more frequent among children whose parents were married. Family characteristics are known to exert an influence on the oral health of children ^20^. According to the perception of parents/guardians, the treatment of TDI in children eight to ten years of age reduces the impact on oral health-related quality of life. This awareness on the part of the family is very important ^5^. Single parents/guardians have a larger burden of responsibilities and may be more likely to postpone treatment for TDI in their children. Moreover, parents/guardians who spend the day working outside the home are less likely to perceive the occurrence of TDI in their children ^12^.

The use of electronic devices by children was a predictor of seeking treatment for TDI. The increase in the use of electronic devices by children has been the target of investigations due to the impacts such use can have on the health of children and adolescents ^9^. Access to information through the internet can assist in daily activities and the internet is an important resource for the acquisition of knowledge on health and self-care ^9^. It also enables consulting the offer of oral health services and seeking health care providers closer to the home. The present results may indicate that electronic devices with internet access constitute an interactive channel that can be used by pediatric dentists for the prevention of TDI and health education with an emphasis on seeking dental care for immediate treatment or follow-up.

Adequate oral health literacy on the part of parents/guardians was a predictor of seeking treatment for TDI. Similar results were found for adolescents and children about the use of dental services ^11^. Previous studies revealed that low OHL results in a lower likelihood of seeking care **whereas higher** OHL is related to a greater interest in seeking information and greater awareness generated by the acquired knowledge, with attitudes that favor health and seeking health care services ^11^. This is the first study to analyze the influence of the OHL of parents/guardians on seeking treatment for TDIs among children in this phase. The finding is relevant to administrators and pediatric dentists to increase OHL programs directed at the parents/guardians of children eight to ten years of age in settings such as schools and primary care units.

Children who attended private schools were more likely to seek treatment for TDI. Type of school is a socioeconomic indicator in Brazil ^23^ and low socioeconomic levels are related to a poorer health status in children ^20^. Thus, this predictor of seeking treatment reveals the disparity between privileged and underprivileged children ^23^. Another contextual predictor of seeking treatment was the number of primary care units with an oral health team in the school district. In Brazil, primary care units have oral health teams, but coverage is not 100% in all Brazilian cities these oral health teams work with a large demand of people but small offer of appointments (24), which hinders access to oral health services in the population that most needs this type of service. Such services are also responsible for prevention and oral health promotion actions besides treatment procedures for the population in the surrounding neighborhood.

The findings of the present investigation underscore the importance of sociodemographic factors about seeking treatment for TDI ^25^ in children in the mixed dentition phase as well as the need for an action strategy directed at families in situations of greater social vulnerability. The actions of the oral health team on the primary care level of the Brazilian public healthcare system should focus on the influence of the characteristics of the family on seeking treatment for TDI. In the actions developed for this specific group, contextual factors may be more effective at reducing social inequalities in the public healthcare system than individual factors alone ^24^. Thus, oral health teams should intensify intersectoral actions and the school should be the main setting for the expansion of prevention and health promotion actions. It is also necessary to qualify healthcare providers on the primary care level and consolidate a care network directed at the treatment and follow-up of traumatic dental injuries in an integral manner and with greater problem-solving capacity. Thus, social disparities related to seeking treatment for TDI in children and the consequences of TDI could be reduced.

The present study has some methodological limitations, such as possible recall bias in the answers to the sociodemographic questionnaire and OHL-AQ. To reduce the risk of bias, care was taken to use validated instruments and diagnostic criteria established in the literature as well as training and calibration exercises. Regarding external validity, the sample selection process ensured a representative study population, which assists in the generalization of the data to similar circumstances and populations. These data can assist administrators and pediatric dentists in developing strategies to raise the awareness of parents/caregivers regarding the importance of seeking treatment for traumatic dental injuries.
